# Ablation of Unilateral Hippocampal GABAergic Neurons: A Novel Mouse Model of Mesial Temporal Lobe Epilepsy With Hippocampal Sclerosis

**DOI:** 10.1002/cns.70772

**Published:** 2026-01-31

**Authors:** Ting Tang, Jinkun Xu, Bin Fu, Chao Geng, Yuhao Li, Yuying Qi, Changkai Hou, Junping He, Yihe Wang, Yumin Luo, Guoguang Zhao

**Affiliations:** ^1^ Department of Neurosurgery Xuanwu Hospital Capital Medical University Beijing China; ^2^ Department of Neurosurgery Children's Hospital of Nanjing Medical University Nanjing China; ^3^ Clinical Research Center for Epilepsy Capital Medical University Beijing China; ^4^ Central Laboratory Xuanwu Hospital Capital Medical University Beijing China; ^5^ Beijing Municipal Geriatric Medical Research Center Beijing China

**Keywords:** hippocampal sclerosis, mesial temporal lobe epilepsy, mice model, targeted GABAergic neuron ablation

## Abstract

**Aims:**

While various animal models of mesial temporal lobe epilepsy (MTLE) exist, a validated model based solely on hippocampal GABAergic interneuron loss is lacking. We aimed to establish and characterize a novel MTLE with hippocampal sclerosis (HS) model by selective ablation of these neurons.

**Methods:**

Using AAV vectors (flex‐DTA or DIO‐taCasp3‐TEVp), we performed unilateral, partial ablation of GABAergic neurons in the dentate gyrus (DG) or CA1 subregions of VGAT‐Cre mice. Spontaneous recurrent seizures (SRS) were monitored by video‐EEG. Behavioral tests and histopathological analyses were conducted to assess comorbidities and HS features.

**Results:**

DG‐ablated mice all developed SRS, whereas 50%–70% of CA1‐ablated mice did. Lethal SRS occurred in around 50% of DG‐ablated mice. Both ablation models exhibited sustained SRS from Days 8–28, distinct from kainic acid model severity in some parameters. The model recapitulated anxiety‐like behaviors, cognitive deficits, and hallmark HS pathology including gliosis, granule cell dispersion, and mossy fiber sprouting.

**Conclusion:**

Selective unilateral hippocampal GABAergic interneuron loss is sufficient to drive chronic epilepsy with HS. This model provides direct causal evidence and a precise platform for investigating MTLE‐HS mechanisms and therapies.

## Graphical abstract

A novel mouse model of mesial temporal lobe epilepsy (MTLE) with hippocampal sclerosis (HS) by unilateral, partial ablation of hippocampal GABAergic neurons is established. The model recapitulates key features of the human condition, including spontaneous recurrent seizures, HS‐like pathology, and associated cognitive deficits. It offers a targeted tool to study epileptogenesis driven by inhibitory neuron loss.

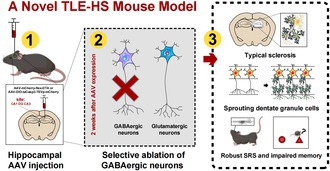

## 1 Introduction

Epilepsy is a group of common neurological disorders characterized by recurrent and unprovoked seizures. Among these, mesial temporal lobe epilepsy (MTLE) is the most common form of drug‐resistant epilepsy, with hippocampal sclerosis (HS) as a prominent pathological hallmark [1]. Recurrent seizure onset in MTLE with HS usually has behavioral comorbidities, including anxiety‐like behaviors and cognitive impairment. The relationship between HS and MTLE is complex, as evidenced by observations of HS without clinical epilepsy and MTLE without typical HS [2]. This study focuses specifically on the prevalent and drug‐resistant MTLE subtype that is associated with HS. A key unresolved question in this specific context is whether the sclerosis itself is a primary driver of the epileptic process or a secondary consequence of it.

HS is distinguished by a range of neuropathological changes, including neuronal loss, granule cell dispersion accompanied by abnormal regeneration, interneuron alterations, gliosis, and mossy fiber sprouting (MFS) [3, 4]. Specially, both disruption and adaptive growth of interneurons have been well characterized in epileptic models [5, 6, 7]. It is noted that absence of GABAergic neurons in the cortex and hippocampus could probably cause SRS in Dlx^−/−^ mice as early as the beginning of the 21st century [8]. There is also an interesting novel finding that human pallial medial ganglionic eminence (MGE)‐type GABAergic interneuron cell therapy could effectively suppress chronic MTLE [9]. It highlighted the question of whether loss of hippocampal GABAergic neurons is sufficient for epilepsy. Notably, a previous study reported that selective immunotoxin‐mediated ablation of hippocampal GABAergic interneurons induced network hyperexcitability and interictal‐like discharges but did not observe the development of spontaneous recurrent seizures (SRS), leaving the causal link between selective interneuron loss and chronic epilepsy unresolved [10].

A variety of animal models have been developed to study MTLE, including chemoconvulsant, electrical stimulation, and genetic approaches, which have significantly advanced our understanding of epileptogenesis and therapeutic strategies [2, 11, 12]. Among these, epileptic seizures in MTLE represent complex pathological network events characterized by an imbalance between excitatory and inhibitory signaling [13, 14]. Kainic acid (KA) is a potent excitotoxic agent renowned for its ability to chemically kill neurons [15]. It has been widely used in experimental mouse models of epilepsy, particularly in scenarios involving chronic spontaneous seizure onset and drug‐resistant epilepsy with hippocampus injection [16, 17, 18, 19]. The KA hippocampal injection model (simplified as the KA model hereafter) is particularly valuable as it mimics the pathophysiological features of human temporal lobe epilepsy, with mechanisms including increasing glutamate release, GABAergic dysfunction, altered ion homeostasis, neuroinflammation, and circuit reorganization [20]. However, the precise mechanism underlying the epileptogenic effects remains unclear. While the KA model reliably induces epilepsy with high penetrance, the timing and frequency of resulting SRS can be unpredictable and irregular across individual animals, a variability that may be influenced by factors such as genetic background [21, 22, 23]. This variability underscores the importance of careful experimental design and interpretation when utilizing the KA model for epilepsy related study.

Diphtheria toxin A (DTA) fragment and taCaspase3‐Tobacco Etch Virus protease (taCasp3‐TEVp) are well‐known inducers of programmed cell death, primarily apoptosis, and are commonly delivered via AAVs in vivo for targeted cell ablation in neurological research [24, 25, 26, 27]. AAVs are small, non‐pathogenic viruses that are commonly used as vectors for gene delivery due to their ability to efficiently transduce a variety of cell types with minimal immune response. Notably, VGAT (Vesicular GABA amino acid transport) stands as a classical genetic marker of GABAergic neurons [28]. The synergistic application of VGAT‐ires‐Cre mice with AAV vectors expressing the double‐floxed inverse orientation (DIO) or flp‐dependent inversion exchange (flex) element offers a robust approach for precisely targeting GABAergic neurons in vivo.

In this research, we developed a new SRS mouse model with hippocampal sclerosis characterized by unilateral partial ablation of hippocampal GABAergic neurons utilizing AAV‐DTA or AAV‐taCasp3‐TEVp. It allows for a thorough investigation into the effects of GABAergic neuron loss and aids in reducing potential confounding factors that contribute to the complex processes involved in the development of epilepsy.

## 2 Methods

### 2.1 Experimental Animals

C57BL/6 mice were procured from Vital River Laboratory Animal Technology, while VGAT‐ires‐Cre (No. 028862) mice sourced from the Jackson Laboratory were generously provided by the Chinese Institute for Brain Research (Beijing, China). Male mice of approximately 8 weeks old and weighing 23–25 g were selected for inclusion in this study. Mice were housed in groups under a 12/12 light/dark cycle, with food and water freely available. The animals were randomly assigned to different groups. All experimental procedures were conducted during the light phase, adhering to the National Institutes of Health guidelines, and the methodology was approved by the Ethics Committee of Xuanwu Hospital.

### 2.2 AAV and Reagents

AAV vectors were packaged with titers of 1–10 × 10^12^ viral genomes (v.g.)/ml, and 200 nL of these vectors were injected into a single site within the target area of the mouse brain for modeling. Specifically, the rAAV2/9‐mCherry‐flex‐DTA (5.5 × 10^12^ v.g./mL, expressing mCherry in all infected cells, simplified as AAV‐flex‐DTA) was obtained from Addgene plasmid (#58536). The rAAV2/9‐DIO‐taCasp3‐TEVp‐mCherry (2.0 × 10^12^ v.g./mL, expressing mCherry only in cre‐positive cells, simplified as AAV‐DIO‐taCasp3‐TEVp) and the rAAV2/9‐DIO‐mCherry (2.0 × 10^12^ v.g./mL) as negative control (NC) were kind gifts from the Chinese Institute for Brain Research. KA (ab144490) was purchased from Abcam, and 100 nL diluted as 2 mg/mL in saline was injected per mouse brain.

### 2.3 Intrahippocampal Microinjection and Skull Electrode Implantation

Mice were anesthetized with 250 mg/kg avertin (i.p.) and securely immobilized in a stereotaxic apparatus (RWD, China). Utilizing a microsyringe pump (Nanoject III, Drummond), the AAV vector or KA was administered through a precisely drilled hole in the skull at a slow velocity of 1 nL/s. To accurately target specific regions of the hippocampus by AAV, the following stereotaxic coordinates (anterior–posterior [AP], medial‐lateral [ML], dorsal‐ventral [DV] from the bregma, in mm) were employed: cornu ammonis 1 (CA1): −2.00, ±1.50, −1.25; dentate gyrus (DG): −2.00, ±1.50, −1.80. As the spread range of KA is uncontrollable and cannot be accurately observed, it was injected into the center region: −2.30, ±1.75, −1.75, following the classical procedure [16]. The side of the hippocampus receiving the injection is referred to as the ipsilateral side, while the other is the symmetrical contralateral side. To avoid potential confounding effects of modeling sequence, every three consecutive mice undergoing modeling were processed as a randomized triplet containing one mouse from each group.

For mice underwent video electroencephalogram (vEEG) recording, skull electrodes were then inserted and securely fixed using dental cement, following established standards [29, 30]. The electrodes set up by three stainless steel screws (O.D. = 1 mm, lenth = 2 mm, impedance 700–1000 Ω) were securely fixed to the skull, including a single point monitoring electrode around the skull surface of the hippocampus projection (the central cranial planar projection coordinates: AP‐2.50, ML ±2.50), with one ground electrode and one reference electrode (the central cranial planar projection coordinates: AP‐7.00, ML ±2.00). These screws were then entwined by wrapped silver wires (Cat No. 786000, A‐M Systems) connected to the needle of the row pins (No. 2305281801, Kedou Brain‐Computer Technology Co. Ltd.) with secure fixation. For mice without EEG monitoring, the scalp of the mouse was stitched properly after injection.

### 2.4 vEEG Recordings and Analyses

Following the insertion of skull electrodes, mice were connected to the NeuSen H signal acquisition system (Neuracle, Changzhou) for 24‐h continuous monitoring, commencing on the 8th day post‐surgery and continuing for the subsequent 3 weeks. To assess seizure severity, the revised Racine (r‐Racine) scale (−1 to 7) was employed, where a tonic–clonic seizure with continuous spike–wave discharges was scored as 5 (when the mouse lay on its belly) or 6 (when it lay on its side or exhibited wild jumping) and was assigned a score of 7 if animal death happened several minutes after seizure onset [31]. The onset and termination of seizures were precisely determined based on the EEG signal, with the EEG filter settings adjusted to 1–70 Hz and the notch filter activated.

### 2.5 Open‐Field Test (OFT) and Novel Object Recognition Test (NORT)

OFT and NORT were designed to evaluate anxiety‐like behaviors and recognition memory in animal models, respectively. We used these two behavioral tests here to assess cognitive and emotional functions within the inter‐ictal periods in epilepsy models following standard procedures [32]. A test arena measuring 40 cm × 40 cm × 30 cm was utilized for both tests. In OFT, mice are acclimatized to the test arena for 30 min prior to the test for habituation. Then, a single mouse is placed in the center of the arena and allowed to explore freely for 10 min. Data including locomotor activity (total distance traveled) and anxiety‐like behavior (time spent in the central zone) were collected. While in NORT, mice were allowed to explore the empty arena for 10 min on Day 1. Then, on Day 2, a single mouse was allowed to freely explore the arena with two identical objects (A1 and A2) symmetrically placed for 10 min. Exploration is defined as sniffing, touching, or directing the nose toward the object within ≤ 2 cm. On Day 3, the familiar object (A1) is replaced with a novel object (B), and exploration time for each object is recorded during 10‐min tests. The discrimination index (DI) was calculated as DI = (*T*_novel_ − *T*_familiar_)/(*T*_novel_ + *T*_familiar_).

### 2.6 Immunofluorescence (IF) Staining

On the 28th day following the modeling, mouse brain tissues were meticulously collected under standard procedures following tissue perfusion. The specimens were fixed in 4% formalin and subsequently dehydrated in 30% sucrose solutions. Frozen sections of the brain tissue (thickness: 30 μm) were prepared for subsequent immunofluorescence staining assays. Following permeabilization and blocking, specific primary antibodies were employed: neuronal nuclei antigen (NeuN, ab177487, Abcam, 1:500), glial fibrillary acidic protein (GFAP, ab53554, Abcam, 1:1000), Iba1 (ab5076, Abcam, 1:500), VGAT (131004, Synaptic Systems, 1:500), vesicular glutamate transporter (VGLUT, 135011, Synaptic Systems, 1:500), Synaptoporin (102005, Synaptic Systems, 1:500), and Doublecortin (DCX, 4604S, CST, 1:300). Subsequently, the corresponding fluorescent secondary antibodies were applied, and DAPI was utilized for nuclear staining.

Images were acquired utilizing the Mica Microsystem (Leica). Confocal images of z‐stack scans (15 μm depth, 1.2 μm step) were generated for mossy fiber staining. The images were imported into ImageJ software for quantitative analysis, according to the standard procedures [33, 34]. Data collected from at least six mice were analyzed for each experiment. For each mouse, three brain sections were used, and for each section, at least two images of the target area under the 40× objective lens were calculated. The target brain areas were delineated manually, followed by the calculation of surface area and cell counting. For immunofluorescence intensity calculation, the positive signal ratio (%) = positive signal area/total area × 100. For cell counting, the cell density = the cell number/the target brain area. All positive fluorescence ratio and cell counting assessments were conducted manually based on the fluorescent spot automatic detection method in ImageJ by two technicians blinded to the grouping conditions, and the average values were subsequently compiled.

### 2.7 RNAscope In Situ Hybridization

RNAscope multiplex fluorescent in situ hybridization was performed using the RNAscope Multiplex Fluorescent Reagent Kit v2 (Advanced Cell Diagnostics) according to the manufacturer's protocol. Brifly, fixed‐frozen brain sections (20 μm) were fixed in 4% PFA and then dehydrated. Sections were treated with hydrogen peroxide, followed by target retrieval and protease digestion. Probes targeting mouse *Slc32a1* (VGAT, Cat #319191) and *Slc17a7* (VGLUT1, Cat #501101) were hybridized simultaneously. Signal amplification and development were performed using Opal 520 and Opal 570 fluorophores (Akoya Biosciences), respectively. Sections were counterstained with DAPI. Images were acquired using a confocal microscope (Leica TSC SP8). The numbers of VGAT‐positive and VGLUT1‐positive neurons within the hippocampal subregions of interest were quantified using ImageJ software by an investigator blinded to the experimental groups.

### 2.8 Fluoro‐Jade C (FJC) Staining

FJC staining was performed to assess neuronal degeneration following interventions according to the manufacturer's protocol (BSS‐TR‐100‐FJT, Biosensis). Briefly, fixed‐frozen brain sections (20 μm) were mounted on gelatin‐coated slides, dried, and then immersed in a basic alcohol solution (1% NaOH in 80% ethanol) for 5 min. After rinsing, sections were incubated in 0.06% potassium permanganate for 2 min, followed by thorough washing. Subsequently, sections were stained with a Fluoro‐Jade C and DAPI mixed solution for 10 min. After washing, slides were dried, cleared in xylene, and coverslipped with DPX mounting medium. FJC‐positive neurons were visualized and imaged using a fluorescence microscope (excitation 480 nm, emission 525 nm, Leica TSC SP8).

### 2.9 Data Analysis

All the experiments were performed in at least six independent biological replicates. EEG filtering analysis was completed under the EEGlab module using Matlab software. Sample sizes (n) represent the number of mice utilized, as detailed in the figure legends. GraphPad Prism 8.0 Software was used for statistical analysis and graphing. Differences between the two groups were statistically analyzed using Student's *t*‐test, while multiple comparisons were assessed using one‐way ANOVA followed by Tukey's test for pairwise comparisons. Data are presented as the mean ± SD. For the analysis of the time to first SRS (epileptogenesis latency), Kaplan–Meier survival curves were plotted. Differences were considered statistically significant when *p* < 0.05.

## 3 Results

### 3.1 AAV‐mCherry‐Flex‐DTA and AAV‐DIO‐taCasp3‐TEVp‐mCherry Induced Ablation of GABAergic Neurons in VGAT‐Ires‐Cre Mice

**FIGURE 1:**
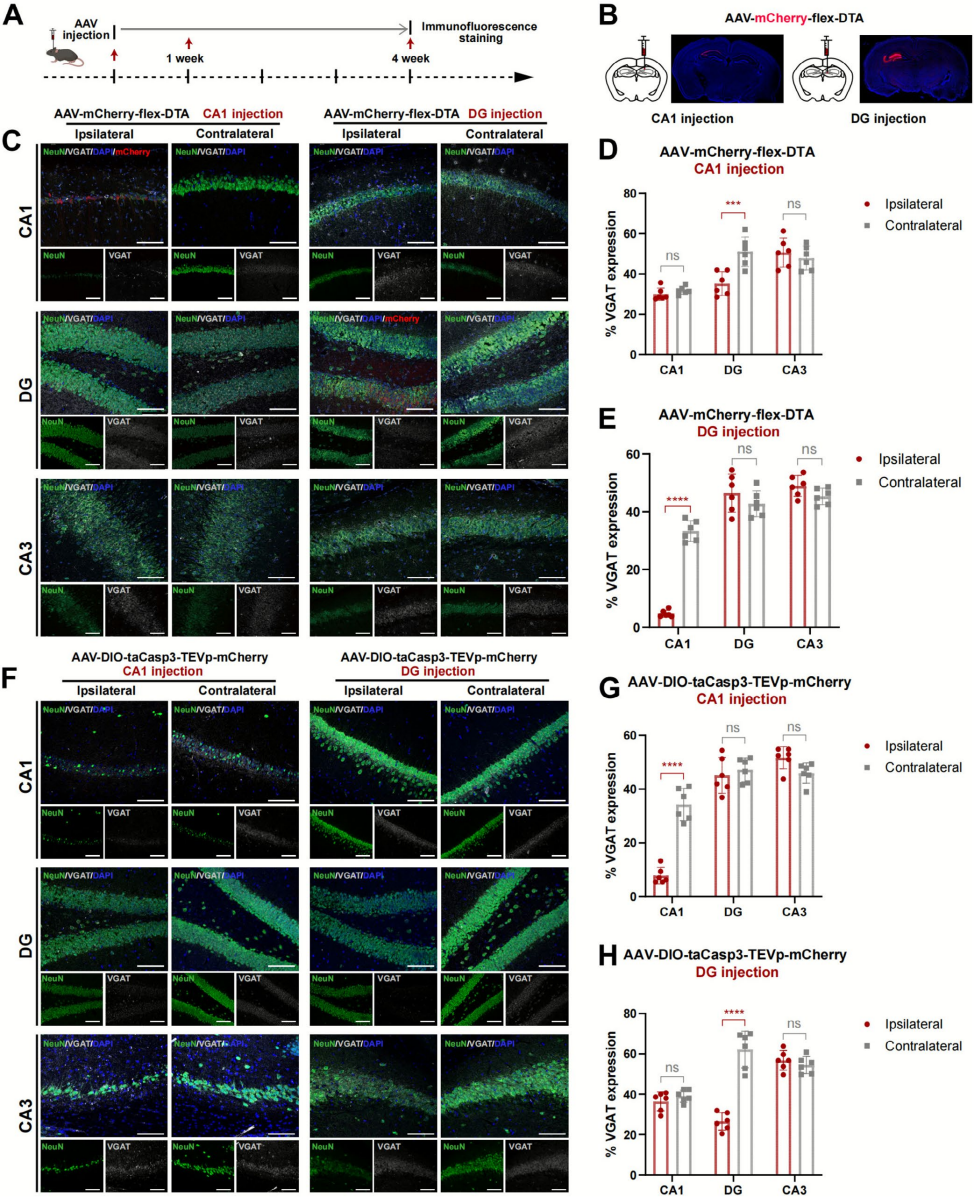
Efficacy of GABAergic neuron ablation via AAV‐mCherry‐flex‐DTA or AAV‐DIO‐taCasp3‐TEVp‐mCherry hippocampal injection. (A) Timeline of the epileptic modeling and the immunofluorescence staining test. (B) Large scan of the whole mouse brain section after AAV‐mCherry‐flex‐DTA injection at the CA1 (left) or DG (right) subregion. (C) Immunofluorescence detection of NeuN, VGAT, and mCherry expression at hippocampal subregions (CA1, DG, CA3) in AAV‐mCherry‐flex‐DTA injection models at CA1 or DG subregion. Scale bar = 100 μm. (D, E) Statistical analysis of VGAT positive signal ratio in (C), for CA1 (D) and DG (E) injection models, respectively. *n* = 6 per group, unpaired two‐tailed Student's t‐test. (F) Immunofluorescence detection of NeuN and VGAT expression at hippocampal subregions (CA1, DG, CA3) in AAV‐DIO‐taCasp3‐TEVp‐mCherry injection models at CA1 or DG subregion. Scale bar = 100 μm. (G, H) Statistical analysis of VGAT positive signal ratio in (F), for CA1 (G) and DG (H) injection models, respectively. *n* = 6 per group, unpaired two‐tailed Student's *t*‐test. Data are presented as mean ± SD. ***p < 0.001, ****p < 0.0001, ns means not significant. VGAT, vesicular GABA transporter.

A Cre‐dependent AAV, AAV‐mCherry‐flex‐DTA or AAV‐DIO‐taCasp3‐TEVp‐mCherry, was injected into the hippocampus of VGAT‐ires‐Cre transgenic mice for cell‐specific ablation of GABAergic neurons for modeling. At the 4‐week mark, mice were euthanized to assess the efficacy of GABAergic neuron ablation by IF staining (Figure 1A). In the AAV‐mCherry‐flex‐DTA injection group, the infection area was delineated based on mCherry expression (Figure 1B). The results revealed a significant loss of GABAergic signals within the injection infiltration zone of AAV‐flex‐DTA (Figure 1C–E) and of AAV‐DIO‐taCasp3‐TEVp (Figure 1F–H), compared to the contralateral side of the same subregions (*p* < 0.001). However, there was no change of VGAT signal in the non‐injection areas (*p* > 0.05, Figure 1C–H). Also, loss of GABAergic neuron bodies in AAV‐DIO‐taCasp3‐TEVp injected models was verified by RNAscope (Figure S1A,B). The NC without intervention were provided as a reference, with no obvious difference from that on the contralateral side of GABAergic ablation (Figure S2A,B).

In the KA model, ablation of GABAergic neurons was most prominent in the ipsilateral CA1 region (*p* < 0.0001), while no significant changes were detected in the ipsilateral DG, CA3, or any contralateral region (*p* > 0.05, Figure S2A,B). It was concluded that the ablation of GABAergic neurons by AAV is consistent with the injection site, while the loss of GABAergic neurons in the KA model had its unique spatial selectivity.

### 3.2 Ablation of Hippocampal GABAergic Neurons by AAV Expressing DTA or taCasp3‐TEVp Induced SRS

**FIGURE 2:**
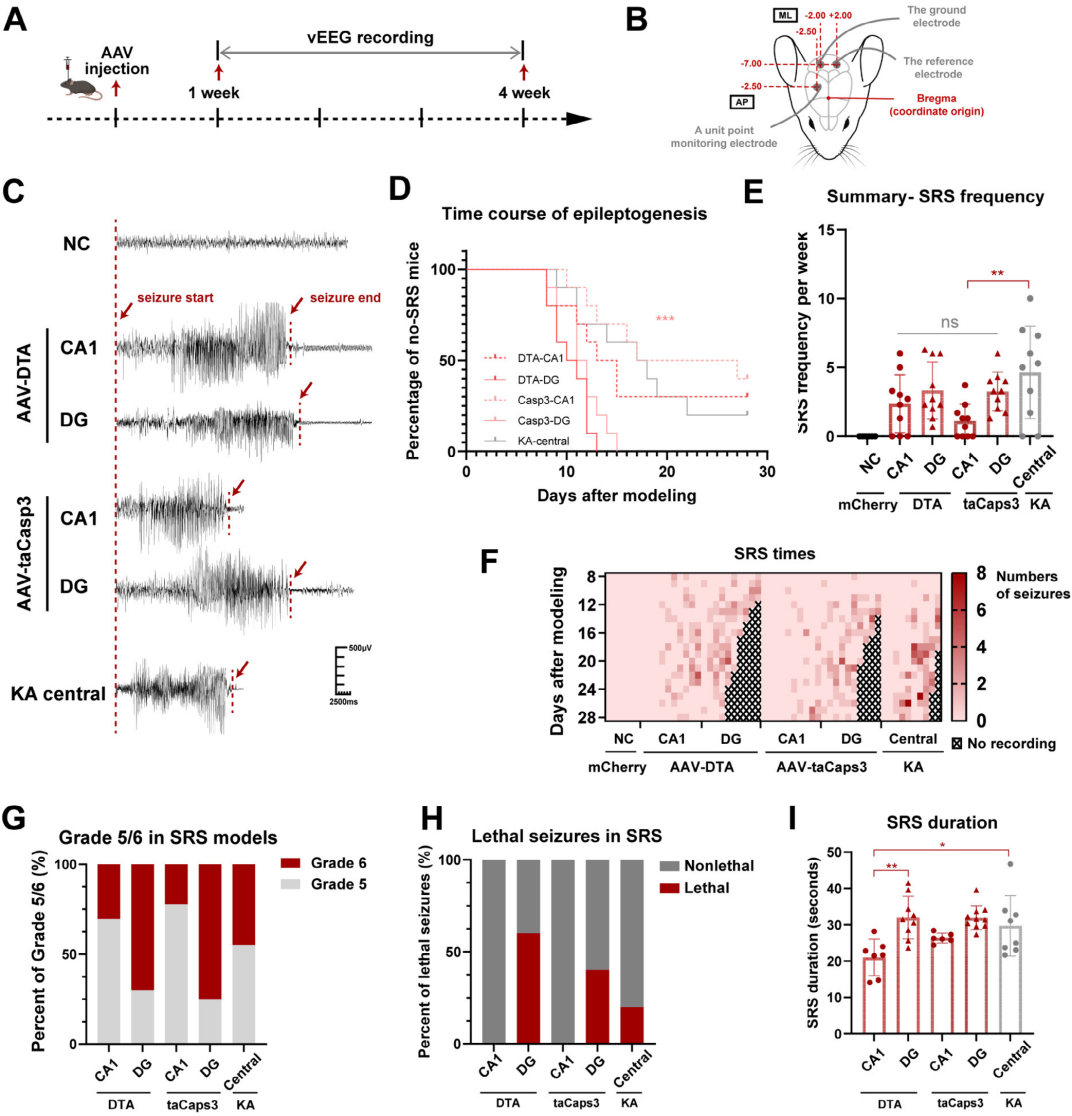
SRS characteristics induced by hippocampal GABAergic neuron ablation. (A) Timeline of the vEEG recording during 1‐ and 4‐week after modeling. (B) Schematic of the positions where electrodes were inserted in the mouse brains. (C) Representative EEG tracings depicting the interictal period and SRS onset in hippocampal GABAergic neuron ablation models (AAV‐mCherry‐flex‐DTA and AAV‐DIO‐taCasp3‐TEVp‐mCherry) and KA injection models. (D) The epileptogenic time course of each group. *n* = 10 for each group. There is a significant difference in comparison of DTA‐CA1 versus DTA‐DG (*p* < 0.05), DTA‐DG versus Casp3‐CA1 (*p* < 0.001), DTA‐DG versus KA‐ central (*p* < 0.01), Casp3‐CA1 versus Casp3‐DG (*p* < 0.01), and Casp3‐DG versus KA‐ central (*p* < 0.01), with no significant difference between the other two groups. (E) Summary of the weekly SRS frequency for each group highlighting the mean SRS rate and differences between groups. *n* = 6 for NC group, *n* = 10 for each other group, one‐way ANOVA followed by Tukey's test. (F) Heat map visualization of the daily SRS frequency for each mouse during the post‐injection period of AAV from Days 8 to 28. No recordings marked after mice dead. (G) Percentage of SRS events classified as Racine Grade 5 and Grade 6 during the recording period for each group. *n* = 10 per group. (H) Percentage of lethal seizures during the recording period for each group. *n* = 10 per group. (I) Summary of the mean seizure duration for each mouse in each group. *n* = 7 for CA1 in AAV‐DTA group, *n* = 6 for CA1 in AAV‐taCasp3 group, and *n* = 10 for each other group, one‐way ANOVA followed by Tukey's test. Data are presented as mean ± SD. *p < 0.05, **p < 0.01, ***p < 0.001, ns means not significant. KA, kainic acid; NC, negative control; SRS, spontaneous recurrent seizures; vEEG, video electroencephalogram.

Hippocampal GABAergic neuron ablation mice were monitored using 24 h/7 d EEG recordings during the 8th and 28th day after modeling (Figure 2A), with the schematic of the electrode position shown in Figure 2B. A total of 6 mice in the sham group and 10 mice in each experimental group were included (Table S1). Under vEEG recording, mice exhibited spontaneous tonic–clonic seizures with high‐amplitude polyspikes and spike–wave discharges (Figure 2C). To assess the epileptogenic time course and severity, we evaluated both the latency to first seizure and several parameters of established epilepsy. Kaplan–Meier analysis revealed the pattern of seizure onset in all ablation groups, with the CA1‐targeted groups, particularly taCasp3‐CA1, exhibiting a relative delay and milder phenotype (Figure 2D). Once established, the mean frequency of SRS ranged from 1.10 to 3.33 times per week, demonstrating no significant variation among different hippocampal ablation regions (*p* > 0.05, Figure 2E). While a lower SRS frequency was only exhibited in the taCasp3‐CA1 model compared to the KA model (*p* < 0.01, Figure 2E).

The overall effects of the DTA model and the taCasp3 model were comparable, with the latter group showing a seemingly weaker SRS severity, although the difference was not statistically significant (*p* > 0.05, Figure 2F–I). Regarding the differences among ablation regions, the SRS severity of the CA1 group was generally weaker, characterized by a lower seizure frequency (Figure 2E,F), a lower Grade 6 seizure ratio (Figure 2G), no lethal seizures (Figure 2H), and a slightly shorter duration (Figure 2I). Notably, the CA1 model stood out with approximately 50% of mice exhibiting no SRS (Figure 2F). The overall SRS pattern in the KA model was intermediate compared to each group of hippocampal GABAergic neuron ablation (Figure 2F–I). In a word, it was found that GABAergic neuron ablation led to chronic SRS, comparable to KA models.

### 3.3 Hippocampal GABAergic Neurons Ablation Induced Anxiety‐Like Behaviors and Impaired Recognition Memory

**FIGURE 3:**
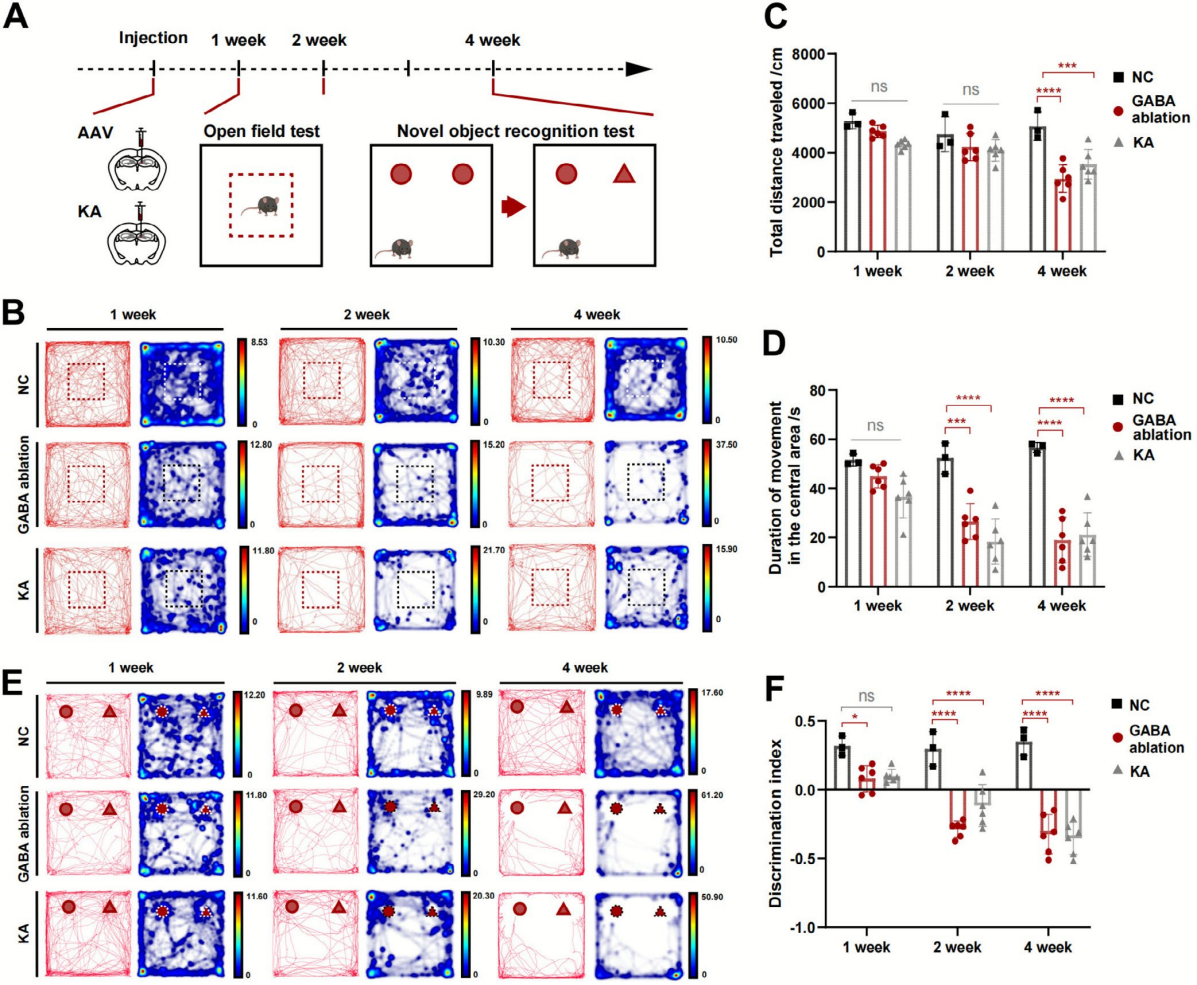
Anxiety‐like behaviors and cognitive impairment assessment of the GABAergic neuron ablation model in mice. (A) Timeline of behavioral experiments including the open field test and the novel object recognition test conducted at 1, 2, and 4 weeks post‐modeling. (B) Locomotor trajectories and movement frequency during the open field test in each group at 1, 2, and 4 weeks post‐modeling. (C) Statistical analysis of total distance traveled by mice during the open field test at each time point. *n* = 3 for the NC group, *n* = 6 for the other groups, one‐way ANOVA followed by Tukey's test. (D) Statistical analysis of time spent exploring the central area of the open field at each time point. *n* = 3 for the NC group, *n* = 6 for the other groups, one‐way ANOVA followed by Tukey's test. (E) Locomotor trajectories and movement frequency in the novel object recognition test at 1, 2, and 4 weeks post‐modeling. (F) Discrimination index (DI) of each group during the novel object recognition test at each time point. *n* = 3 for the NC group, *n* = 6 for the other groups, one‐way ANOVA followed by Tukey's test. Data are presented as mean ± SD. *p < 0.05, ***p < 0.001, ****p < 0.0001, ns means not significant. KA, kainic acid; NC, negative control.

Memory impairment associated with anxiety disorders is a characteristic of MTLE with HS. To further investigate this phenotype of the GABAergic neuron ablation model based on AAV‐DIO‐taCasp3‐TEVp injection in the DG subregion, we conducted the OFT and the NORT in mice at the timepoints of 1‐, 2‐, and 4‐weeks post induction (Figure 3A). The mice with AAV‐DIO‐mCherry injection in the DG were used as the NC group.

Analysis of the locomotor trajectories and activity frequency during the OFT indicated that our GABAergic neuron ablation model did not show significant differences compared to the control group at 1‐ and 2‐week (Figure 3B,C). However, substantial reductions in movement trajectories were observed at the 4‐week time points (Figure 3B,C), similar to what was observed in the KA models. Furthermore, the time spent exploring the central zone of the arena was assessed, as longer stays typically reflect increasing anxiety‐like behavior. Our results indicated no significant differences between the GABAergic neuron ablation and the NC groups at 1‐week, but by weeks 2 and 4, the GABAergic neuron ablation model exhibited significant anxiety‐like behaviors comparable to those observed in the KA model (Figure 3D).

Next, we evaluated recognition memory using the NORT. As early as 1‐week, the GABA ablation model exhibited a significant decrease in the discrimination index (DI), indicative of declining memory abilities. At 2‐ and 4‐week, GABAergic neuron ablation mice demonstrated a preference for familiar objects, reinforcing the fact that memory impairments worsen over the disease process (Figure 3E,F). This result corresponded with findings from the KA model, where declined memory abilities were found from the 2‐week period. All these findings are consistent with the clinical symptoms of patients with MTLE, who experience progressively worsening anxiety and cognitive impairment, thereby suggesting the reliability of the GABAergic neuron ablation model in recapitulating the phenotypes of MTLE.

### 3.4 The Integrity of Glutamatergic Cells Was Preserved in the GABAergic Neuron Ablation Epilepsy Model

**FIGURE 4:**
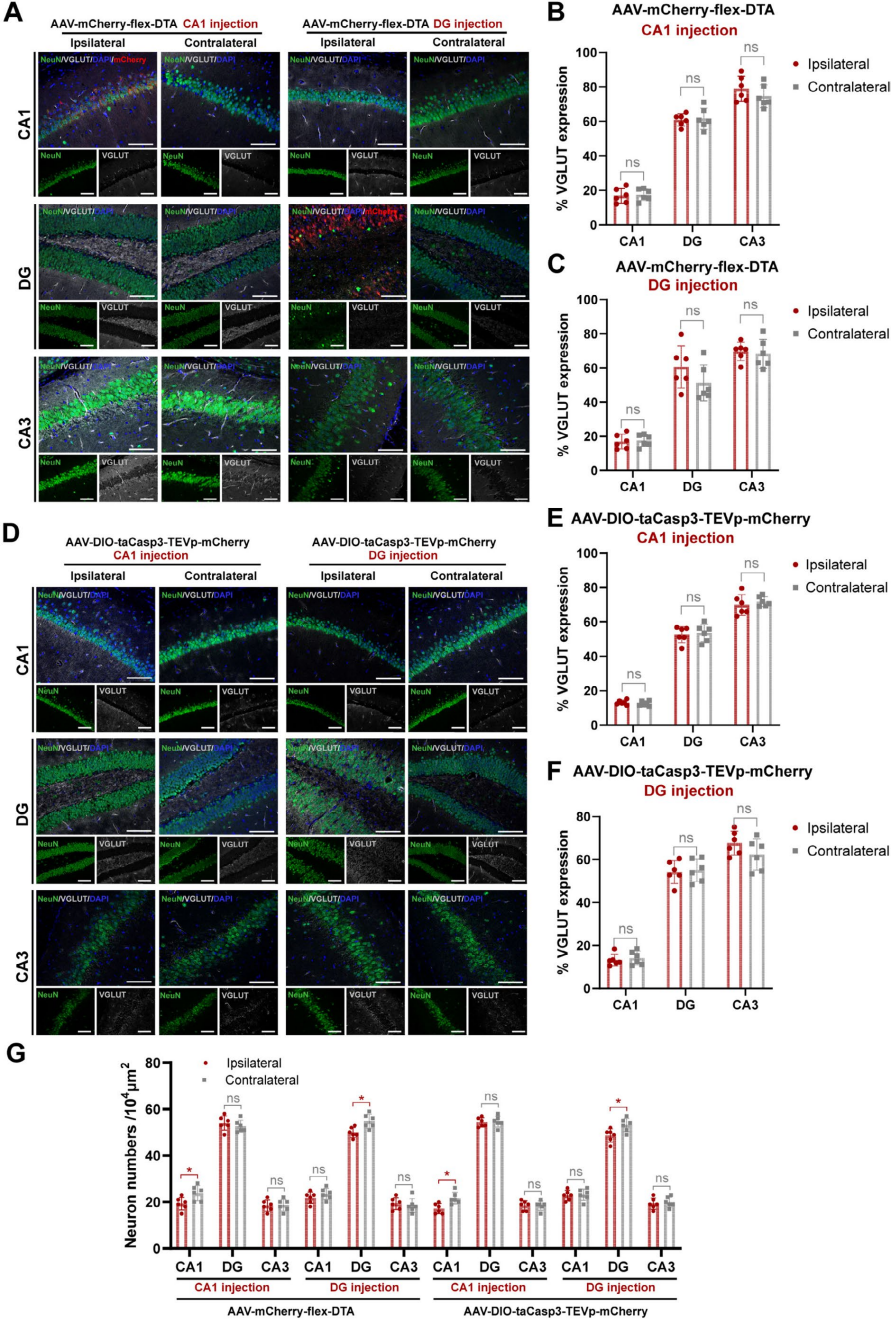
Effect on glutamatergic neurons of GABAergic neuron ablation by AAV‐mCherry‐flex‐DTA or AAV‐taCasp3‐TEVp‐mCherry hippocampal injection. (A) Immunofluorescent labeling of NeuN, VGLUT, and mCherry expression at hippocampal subregions (CA1, DG, CA3) in AAV‐mCherry‐flex‐DTA injection models at CA1 or DG subregion. Scale bar = 100 μm. (B, C) Statistical analysis of VGLUT positive signal ratio in (A), for CA1 (B) and DG (C) injection models, respectively. *n* = 6 per group, unpaired two‐tailed Student's *t*‐test. (D) Immunofluorescent labeling of NeuN and VGLUT expression at hippocampal subregions (CA1, DG, CA3) in AAV‐DIO‐taCasp3‐TEVp‐mCherry injection models at CA1 or DG subregion. Scale bar = 100 μm. (E, F) Statistical analysis of VGLUT positive signal ratio in (D), for CA1 (E) and DG (F) injection models, respectively. (G) Statistical analysis of NeuN positive cell numbers in (A–F). *n* = 6 per group, unpaired two‐tailed Student's *t*‐test. Data are presented as mean ± SD, *p < 0.05, ns means not significant. VGLUT, vesicular glutamate transporter.

To evaluate the alteration of excitatory systems following GABAergic neuron ablation, we quantitatively analyzed VGLUT expression through IF staining. Notably, both GABAergic neuron ablation models, AAV‐flex‐DTA (Figure 4A–C) and AAV‐DIO‐taCasp3‐TEVp (Figure 4D–F), demonstrated preserved glutamatergic signaling integrity throughout hippocampal subregions. Comparative quantification revealed no statistically significant differences in the VGLUT signal between the ipsilateral and the contralateral side (*p* > 0.05, Figure 4C,F). However, there seems to be a trend for a reduction of VGLUT staining in the DG ablation model by AAV‐flex‐DTA injection (*p* = 0.1529, Figure 4C). Also, the integrity of hippocampal glutamatergic neuron bodies in AAV‐DIO‐taCasp3‐TEVp injected models was verified by RNAscope (Figure S1A,C). The alteration of GABAergic and glutamatergic neuron numbers was also evaluated by the statistics of NeuN (Figure 4G).

This selective preservation contrasts markedly with the non‐specific neuronal damage observed in KA models. Histopathological analysis of KA‐treated specimens revealed extensive loss of NeuN positive cells and glutamatergic depletion in injection sites, compared to that in the contralateral side and NC mice (*p* < 0.001, Figure S3). The differential neuropathological profiles underscore the target‐specificity of our GABAergic ablation paradigm, possibly isolating epileptogenic mechanisms from secondary excitatory neurodegeneration. This neuronal degeneration in KA models could also be reflected in the FJC staining (Figure S4A), which is negative in the GABAergic neuron ablation models (Figure S4B).

### 3.5 Selective GABAergic Ablation Triggered Reactive Gliosis in the Hippocampus

**FIGURE 5:**
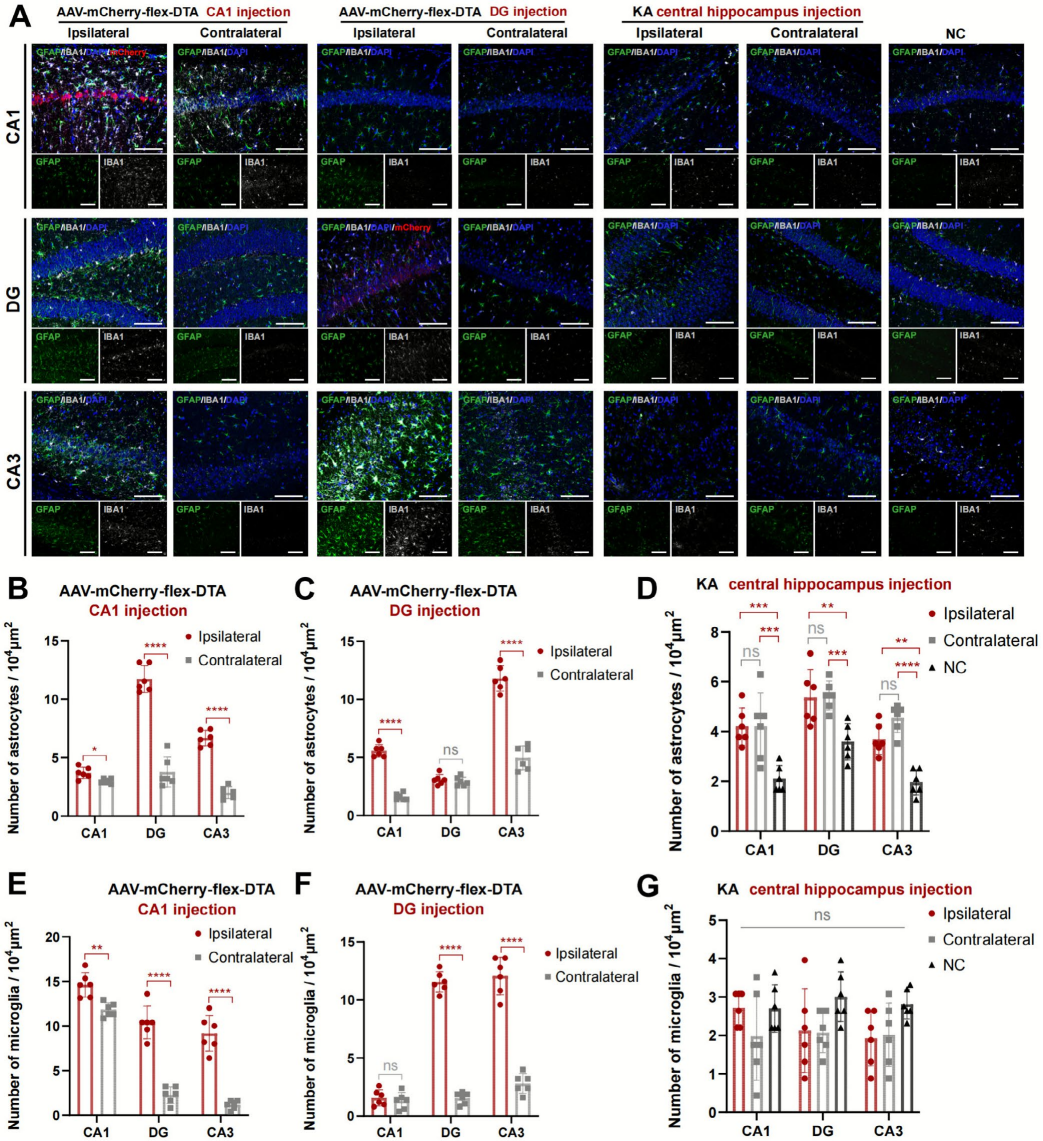
Astrocyte and microglia infiltration in AAV‐mCherry‐flex‐DTA hippocampal‐injection models. (A) Immunofluorescent labeling of GFAP, IBA1, and mCherry expression at hippocampal subregions (CA1, DG, CA3) in GABA ablation and KA models. AAV‐mCherry‐flex‐DTA injected at CA1 or DG, KA at the central hippocampus. Scale bar = 100 μm. (B–G) Statistical analysis of astrocytes and microglia numbers measured by the expression of GFAP and IBA1 in AAV‐mCherry‐flex‐DTA injection models at the CA1 (B, E) or DG (C, F) subregion and KA models (D, G) in (A). *n* = 6 per group, unpaired two‐tailed Student's *t*‐test for pairwise comparison, one‐way ANOVA followed by Tukey's test for multiple group comparisons. Data are presented as mean ± SD. *p < 0.05, **p < 0.01, ***p < 0.001, ****p < 0.0001, ns means not significant. KA, kainic acid; NC, negative control.

As gliosis is one of the classical characteristics of HS, we tested the alteration of glial cells (including astrocytes and microglia) infiltration in the GABAergic neuron ablation models with AAV‐flex‐DTA by IF staining. The glial fibrillary acidic protein (GFAP) and the ionized calcium binding adapter molecule 1 (Iba1) were the classical markers used for astrocytes and microglia staining, respectively. Quantification of canonical marker staining revealed pronounced reactive gliosis, especially within ipsilateral hippocampal subfields (Figure 5A–C,E,F). However, the difference in astrocyte infiltration was not significant in the DG injection subregion, nor microglia infiltration in the CA1 subregion in DG injection models (*p* > 0.05, Figure 5C,F). This gliotic response diverged markedly from the KA‐induced excitotoxic paradigm. In KA models, we observed selective astrocytosis in both ipsilateral and contralateral KA injection sides (*p* < 0.01, Figure 5A,D), without concomitant microglial activation (*p* > 0.05, Figure 5A,G). The dissociation between astroglial and microglial responses highlights pathology‐specific neuroinflammatory signatures.

### 3.6 Ablation of GABAergic Neurons Induced Hippocampal New‐Born Dentate Granule Cells (DGCs) Increased

**FIGURE 6:**
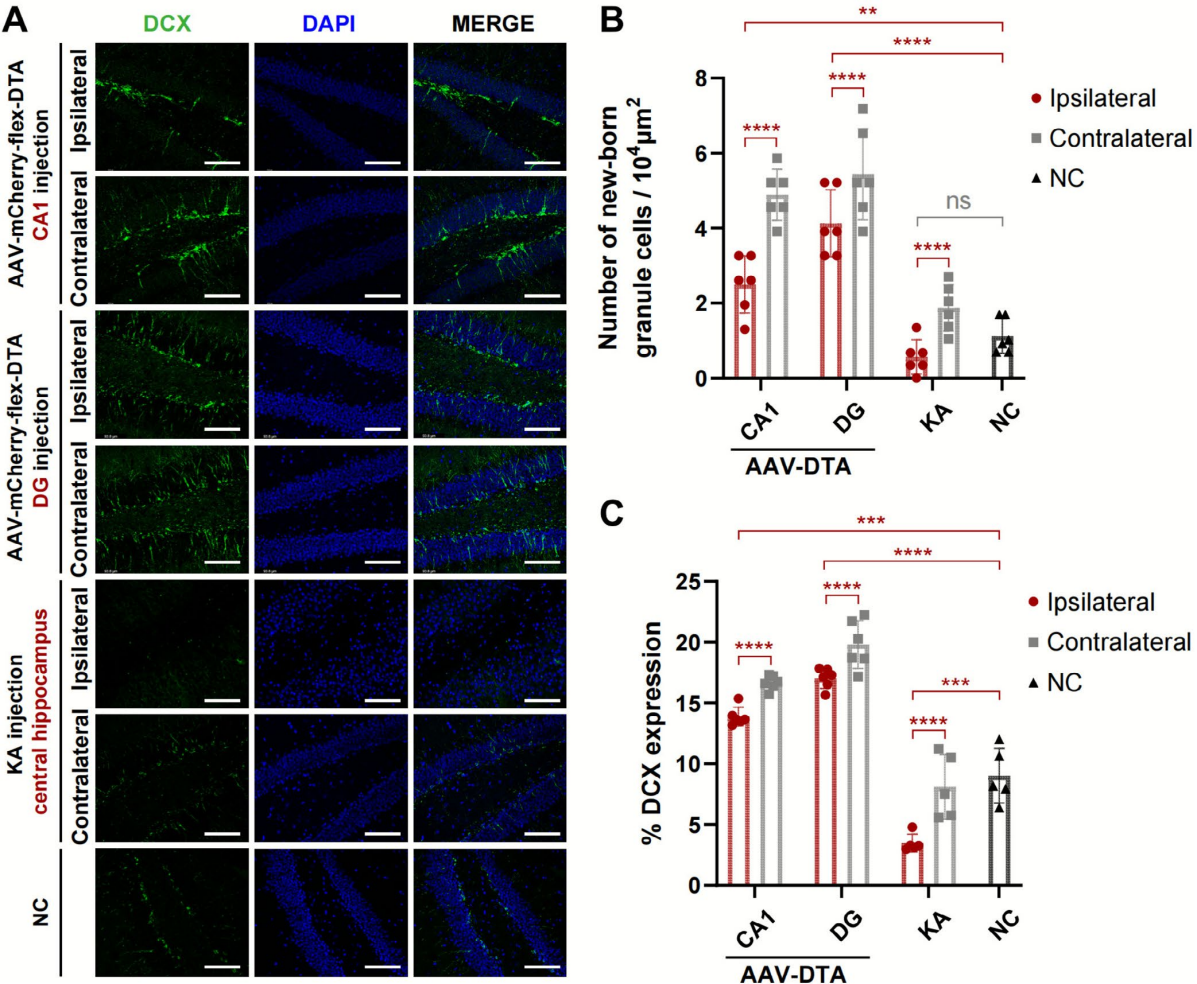
New‐born granule cells increased in AAV‐mCherry‐flex‐DTA hippocampal injection models. (A) Immunofluorescence staining showing the expression of DCX at the DG subregion in the hippocampus in GABA ablation and KA models. AAV‐mCherry‐flex‐DTA injected at CA1 and DG, KA at the central hippocampus. Scale bar = 100 μm. (B) Quantification of newborn granule cell counts in different groups in (A). *n* = 6 per group, one‐way ANOVA followed by Tukey's test. (C) Statistical analysis of DCX positive signal ratio in (A). Groups *n* = 6 per group, one‐way ANOVA followed by Tukey's test. Data are presented as mean ± SD. **p < 0.01, ***p < 0.001, ****p < 0.0001, ns means not significant. DCX, doublecortin; KA, kainic acid; NC, negative control.

DCX, a marker for adult‐generated DGC, was used to quantify cell counts and assess signal percent. Our findings revealed an augmentation in hippocampal neurogenesis within the DG in models of GABAergic neuron ablation. Specifically, regardless of the injection subregion, a notable increase in neurogenesis was observed (*p* < 0.01, Figure 6A–C). Furthermore, it exhibited a higher increase on the contralateral side of injection than the ipsilateral side (*p* < 0.001, Figure 6A–C). However, in KA injection models, a reduction of DGCs in hippocampal neurogenesis was observed specifically on the injection side (*p* < 0.001, Figure 6B,C). Compared to the KA model, the hippocampal regeneration was classically shown in the GABAergic neuron ablation mice.

### 3.7 Ablation of GABAergic Neurons Induced Hippocampal MFS

**FIGURE 7:**
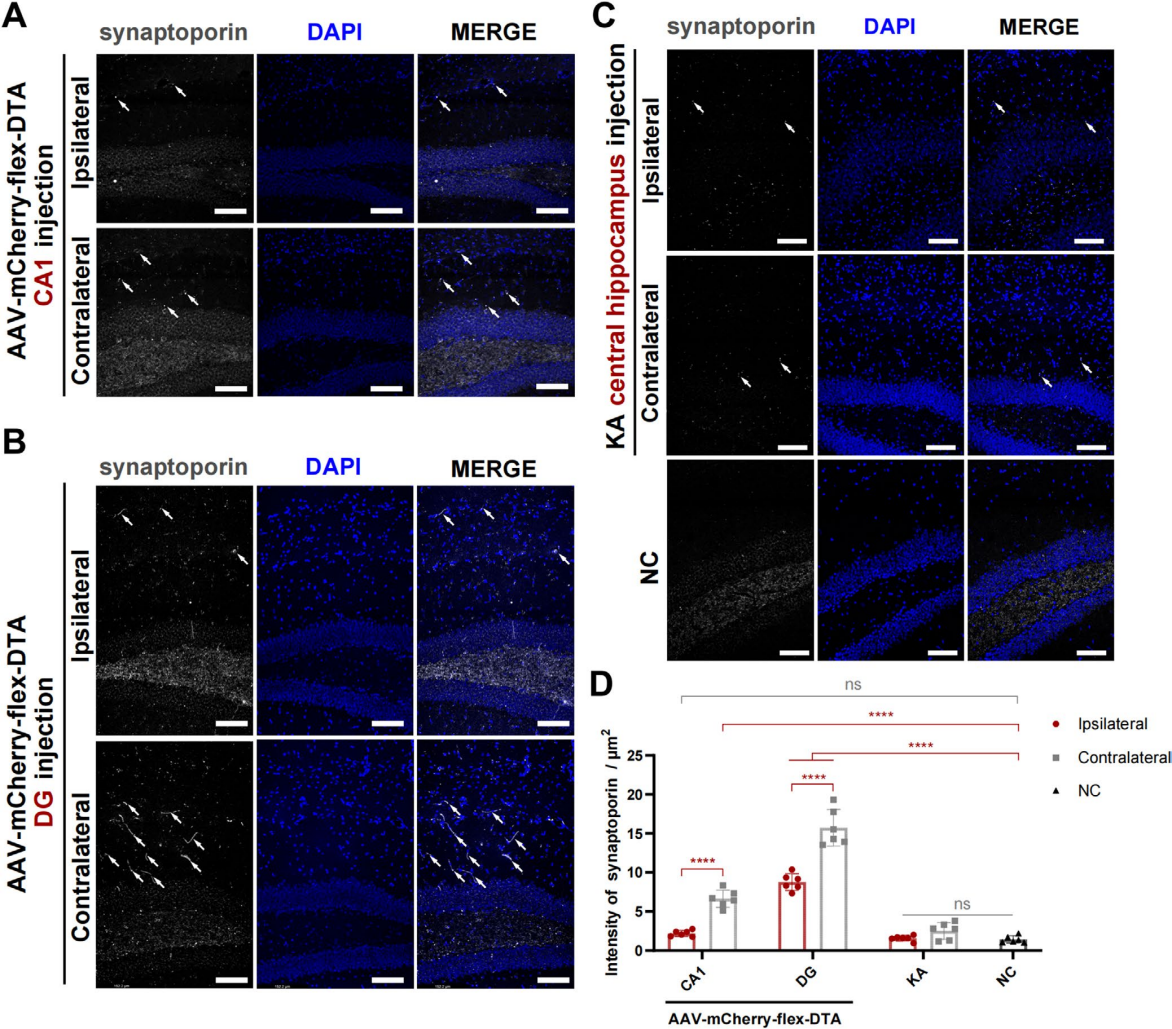
Mossy fibers sprouting in AAV‐mCherry‐flex‐DTA hippocampal injection models. (A–C) Immunofluorescence staining showing the expression of synaptoporin at the DG subregion in the hippocampus in GABA ablation (A, B) and KA models (C). AAV‐mCherry‐flex‐DTA injected at CA1 (A) and DG (B), KA at the central hippocampus (C). Scale bar = 100 μm. (D) Statistical analysis of the expression of synaptoporin in the molecular layer in (A–C). *n* = 6 per group, one‐way ANOVA followed by Tukey's test. Data are presented as mean ± SD. ****p < 0.0001, ns means not significant. KA, kainic acid; NC, negative control.

The anti‐synaptoporin antibody visualizes the axons of granule neurons (mossy fibers), reversely from the cell layer to the molecular layer. Hippocampal MFS was classical on the contralateral side of AAV injection in GABAergic neuron ablation models, compared with both the ipsilateral side and the NC models, wherever the injection site (*p* < 0.0001, Figure 7A,B,D). Notably, MFS on the ipsilateral side was apparent only when GABAergic neurons in the DG region were ablated (*p* < 0.0001, Figure 7B,D). However, in KA models, hippocampal MFS was not observed at the time point tested, and mossy fibers within the polymorphic layer appeared to be disrupted (*p* > 0.05, Figure 7C,D). At the 4‐week time point, sprouting mossy fibers were more obvious in the GABAergic neuron ablation mice than in the KA model.

## 4 Discussion

In the field of neuroscience, it is commonly acknowledged that a deficiency in GABAergic neurons is intricately linked to the occurrence of epileptic seizures, while their activation inversely mitigates the frequency and severity of epileptic episodes [35, 36]. Notably, patients diagnosed with MTLE often exhibit a loss of GABAergic neurons on the epileptic side [14]. However, despite these correlations, it is still unresolved whether their loss is a cause or a consequence of MTLE [37, 38]. The deletion or mutation of some specific genes in the cortical or somatic GABAergic neurons was widely reported to induce epilepsy [39, 40, 41, 42, 43]. However, there is no direct evidence in the previous studies that neural inhibition of hippocampal GABAergic neurons, including GABAergic antagonists and chemogenetics, could induce tonic–clonic seizure onset, till now [44, 45]. Our current study presents novel findings, marking the first instance where HS‐like characteristics have been observed in epileptic mouse models induced by the targeted ablation of unilateral partial hippocampal GABAergic neurons. This discovery suggests a potential pathological mechanism underlying the development of epilepsy, particularly in the context of GABAergic neuron loss.

Our findings both converge with and extend beyond prior approaches to model GABAergic dysfunction in epilepsy. The work of Antonucci et al. [10], using immunotoxin‐mediated ablation of hippocampal GABAergic interneurons, reported network hyperexcitability but did not observe SRS. This apparent phenotypic difference may stem in part from methodological distinctions, whereas the EEG assessment in that study was limited to 1‐h daily sessions [10]. However, our study employed continuous 24‐h monitoring, which is substantially more capable of capturing sporadic SRS. Interestingly, the VGLUT1 excitatory afferents were unaffected in their study, which is basically consistent with our results. In another prior investigation, targeted ablation of GABAergic neurons in the unilateral hippocampal CA1 region of glutamic acid decarboxylase 2 (GAD2)‐Cre mice also failed to elicit sustained epilepsy, with SRS lasting only for a few days [37]. It is important to note that GAD2 is neither sufficient nor necessary for defining GABAergic identity [46], and its expression has also been reported in certain excitatory neuronal populations, implying possible off‐target effects in such a model [47, 48].

In contrast, our current research demonstrates that epileptic SRS persisted for at least 1 month in models with VGAT‐expressing GABAergic neuron ablation. VGAT is the single form of GABA transporter, and no cross‐expression has been found in other cells yet. This characteristic makes the result of our study more explainable. However, the immunostaining of GABAergic neurons is difficult, and we have failed to achieve an efficient and specific staining effect after trying several antibodies. Fortunately, the final performance with anti‐VGAT antibody was consistent with the previous study [34], and the percentage of the positive area could represent the number of GABAergic neurons. Better marks of GABAergic neurons may help in this field. Additionally, it is worth noting that in our study, the ablation of GABAergic neurons in the CA1 region exhibited the least severe effects compared to other hippocampal subregions, thereby presenting a subtlety that might have been challenging to detect. The less severe epilepsy may be related to the reduced expression of recurrent collaterals in CA1 due to GABAergic ablation [49, 50]. These observations underscore the complexity of GABAergic neuron function in epilepsy and motivate further investigations to elucidate the underlying mechanisms.

Intriguingly, our finding aligns with a recent study, where epileptic SRS with HS was observed in rat models following the ablation of bilateral GABAergic neurons via Stable Substance P‐saporin hippocampal injection [51]. Although Substance P was found to be a neuropeptide distributed in all hippocampal GABAergic neurons [52], the neurokinin (NK)‐1 receptor was more broadly distributed in principal cells [53] and many other brain regions [54]. So, Substance P exogenously supplemented could not target GABAergic neurons with high possibility. It may partially explain why bilateral hippocampal injection was necessary in this model. However, unilateral hippocampal GABAergic neuron ablation by AAV was sufficient for SRS alongside HS in our study.

Compared with the GABAergic neuron ablation model newly raised in this study, seizures in the KA hippocampal‐injection model seemed to be stronger with higher frequency. Since the infiltration range of the compound is large, it is difficult to limit the effect to any subregions, and the effect of different injection sites was similar (data not shown). Early studies have pointed out that KA injection through intraventricular injection selectively induced GABAergic neuron death by excitotoxic processes [55, 56]. However, 30% pyramidal cell loss in CA1 was identified in KA hippocampal injection models [20], which is consistent with our results, although the percentage may be higher in our study, probably due to the limited calculation area. So, the epileptogenic mechanism of KA may be complex, and both GABAergic and glutamatergic signals could be impaired based on the evidence currently available.

In addition, it was verified that the GABAergic neuron ablation models developed anxiety‐like behaviors and cognitive impairment with the increasing seizure onset. This phenomenon could also be seen in both KA hippocampal injection mice models and patients with MTLE [57, 58]. The development of cognitive impairment in individuals with epilepsy arises from the complex interplay of multiple pathogenic factors. Notably, recurrent epileptic activity exerts a particularly pronounced detrimental impact on hippocampal integrity, inducing both structural abnormalities and functional disturbances that ultimately impair neuroplasticity and hinder cognitive processes such as memory consolidation and learning capacity [58]. It is critical evidence that our model effectively simulates clinical MTLE characteristics.

Glial infiltration, adult‐born DGC increasing and long‐lasting MFS are the classical pathological characteristics of epileptic HS [20]. In our study, both astrocyte and microglia infiltration could be observed in GABAergic ablation models, while only astrocyte infiltration is significant in KA models in the timepoint of 1 month after modeling. It is interesting that the dynamic changes of microglia in epilepsy models induced by KA exist, in both numbers and morphology [59]. Microglia infiltration was observed within 1 week after modeling in the previous research [59, 60, 61], and the results of our study added at least partial recovery after 1 month.

Our GABAergic neuron ablation model also exhibits increased new‐born DGCs and continuous MFS, especially on the contralateral side of AAV injection. The DCX antibody detects total doublecortin protein, which is highly expressed in newborn neurons and decreases as they mature [62, 63, 64]. This allows DCX to be a valuable marker for identifying adult‐born dentate granule cells in our epilepsy model. While new‐born DGCs have been targeted for chemoconvulsant models [65, 66, 67], seizure‐free outcomes remain elusive, suggesting other mechanisms play crucial roles. Our findings indicate that GABAergic neurons may negatively regulate DGC proliferation, an intriguing hypothesis that merits further exploration.

With the increasingly deeper understanding of HS, axonal reorganization exists not only in dentate granule cells but also is found in interneurons and other excitatory neurons [68]. However, the IF staining by synaptoporin antibody targeting a synaptic vesicle membrane protein enriched in mossy fibers is still an easy and classical method for the detection of axonal reorganization in MTLE [69, 70]. Previous literature indicates that MFS can be observed within 3 weeks post‐modeling in the KA mice model [71]. However, there is a lack of evidence for its persistence over a longer duration, although the variability of KA‐induced pathology is high [71]. In contrast, our findings demonstrate that MFS is not surely detectable 1 month after KA injection under our conditions, while a significant presence of MFS is observed in the GABAergic neuron ablation model. This discrepancy may suggest differences in the underlying mechanisms between these models, warranting further investigation. Interestingly, the MFS seems more classical on the contralateral side of AAV injection. It may suggest the MFS as the result of epilepsy, rather than the cause, which remains verified.

The previous study highlights the varying functions of GABAergic neurons across epilepsy subtypes, focusing primarily on neocortical interneurons [13, 72]. Interestingly, both the inhibition and activation of GABAergic neurons have been linked to epileptogenicity [73, 74], presenting a paradox that demands further investigation. Since different GABAergic neuron subtypes have variable functions, further exploration of each group in epilepsy is crucial. Precise targeting of GABAergic neuron ablation could aid in elucidating the involvement of specific hippocampal subregions in epilepsy pathogenesis.

In summary, our model of unilateral partial ablation of hippocampal GABAergic neurons, which induces spontaneous recurrent seizures along with hippocampal sclerosis, serves as an innovative platform for investigating the consequences of GABAergic neuron loss. This model helps to address and minimize potential confounding factors in the complex processes underlying epileptogenesis and also demonstrates the feasibility of pallial MGE‐type GABAergic interneuron cell transplanting therapy.

## Author Contributions

Ting Tang and Guoguang Zhao proposed the hypothesis. Ting Tang, Jinkun Xu, and Bin Fu did the main part of modeling and data analysis. Bin Fu, Chao Geng, and Yuying Qi provided the modeling of KA hippocampal injection. Ting Tang and Jinkun Xu wrote the main manuscript text and took part in the preparation of all the figures. Guoguang Zhao and Yumin Luo reviewed the statistical methods and led the revision of the manuscript. Yuhao Li, Changkai Hou, Junping He, and Yihe Wang took part in the revision of the manuscript. All authors read the final manuscript.

## Funding

This article received the support of Beijing Natural Science Foundation (Grant No. 7244354), National Natural Science Foundation of China (Grant No. 82201605 and 82030037), National Key R&D Program of China (Grant No. 2021ZD0201801), and Beijing Municipal Health Commission (Grant No. 11000025T000003320606).

## Ethics Statement

This study was approved by the Ethics Committee of Xuanwu Hospital (LYS[2021]118).

## Conflicts of Interest

Yumin Luo is the Associate Editor of *CNS Neuroscience and Therapeutics* and a co‐author of this article. She is excluded from editorial decision‐making related to the acceptance and publication of this article. Editorial decision‐making was handled independently by the other editor to minimize bias. None of the other authors has any conflicts of interest to disclose.

## Supporting information


**Appendix S1:** cns70772‐sup‐0001‐Supinfo.zip.

## Data Availability

The data that supports the findings of this study are available in the Supporting Information of this article.
