# Supplementary material for: Ablation of Unilateral Hippocampal GABAergic Neurons: A Novel Mouse Model of Mesial Temporal Lobe Epilepsy With Hippocampal Sclerosis
Source: CNS Neurosci Ther. 2026 Jan 31;32(2):e70772. doi: 10.1002/cns.70772 (PMC12859687; doi:10.1002/cns.70772)
Supplement: Supplementary file 1 — Appendix S1: cns70772‐sup‐0001‐Supinfo.zip. [file CNS-32-e70772-s001.zip › cns70772-sup-0001-TableS1@Supllementary Table 1.docx]

Supplementary Table 1. Summary of mice injected with AAV for GABAergic neuron ablation (AAV-mCherry for negative control) and KA, the anatomical distribution of mCherry expression, and the total number of seizures observed

| **NO.** | **AAV/ KA** | **Injection**  **site** | **Distribution** | | | **Number of seizures** | **Days recorded** | **Lethal epilepicus** |
| --- | --- | --- | --- | --- | --- | --- | --- | --- |
|  |  |  | **CA1** | **CA3** | **DG** |  |  |  |
| 1 | mCherry | CA1 | +++ | - | + | 0 | 21 | NO |
| 2 | mCherry | CA1 | +++ | - | - | 0 | 21 | NO |
| 3 | mCherry | CA3 | - | +++ | + | 0 | 21 | NO |
| 4 | mCherry | CA3 | - | +++ | - | 0 | 21 | NO |
| 5 | mCherry | DG | - | + | +++ | 0 | 21 | NO |
| 6 | mCherry | DG | + | - | +++ | 0 | 21 | NO |
| 7 | DTA | CA1 | +++ | - | + | 0 | 21 | NO |
| 8 | DTA | CA1 | +++ | - | - | 0 | 21 | NO |
| 9 | DTA | CA1 | +++ | - | - | 0 | 21 | NO |
| 10 | DTA | CA1 | +++ | - | - | 4 | 21 | NO |
| 11 | DTA | CA1 | +++ | - | - | 7 | 21 | NO |
| 12 | DTA | CA1 | +++ | - | - | 8 | 21 | NO |
| 13 | DTA | CA1 | +++ | - | + | 9 | 21 | NO |
| 14 | DTA | CA1 | +++ | - | - | 10 | 21 | NO |
| 15 | DTA | CA1 | +++ | - | - | 15 | 21 | NO |
| 16 | DTA | CA1 | +++ | - | - | 18 | 21 | NO |
| 17 | DTA | DG | - | + | +++ | 2 | 21 | NO |
| 18 | DTA | DG | + | - | +++ | 8 | 21 | NO |
| 19 | DTA | DG | - | - | +++ | 18 | 21 | NO |
| 20 | DTA | DG | - | - | +++ | 19 | 21 | NO |
| 21 | DTA | DG | - | - | +++ | 18 | 16 | YES |
| 22 | DTA | DG | - | - | +++ | 11 | 14 | YES |
| 23 | DTA | DG | - | - | +++ | 4 | 9 | YES |
| 24 | DTA | DG | + | + | +++ | 6 | 7 | YES |
| 25 | DTA | DG | + | - | +++ | 7 | 5 | YES |
| 26 | DTA | DG | - | - | +++ | 7 | 4 | YES |
| 27 | taCasp3 | CA1 | / | | | 0 | 21 | NO |
| 28 | taCasp3 | CA1 |  |  |  | 0 | 21 | NO |
| 29 | taCasp3 | CA1 |  |  |  | 0 | 21 | NO |
| 30 | taCasp3 | CA1 |  |  |  | 0 | 21 | NO |
| 31 | taCasp3 | CA1 |  |  |  | 2 | 21 | NO |
| 32 | taCasp3 | CA1 |  |  |  | 4 | 21 | NO |
| 33 | taCasp3 | CA1 |  |  |  | 4 | 21 | NO |
| 34 | taCasp3 | CA1 |  |  |  | 5 | 21 | NO |
| 35 | taCasp3 | CA1 |  |  |  | 7 | 21 | NO |
| 36 | taCasp3 | CA1 |  |  |  | 11 | 21 | NO |
| 37 | taCasp3 | DG | / | | | 4 | 21 | NO |
| 38 | taCasp3 | DG |  |  |  | 10 | 21 | NO |
| 39 | taCasp3 | DG |  |  |  | 12 | 21 | NO |
| 40 | taCasp3 | DG |  |  |  | 12 | 21 | NO |
| 41 | taCasp3 | DG |  |  |  | 13 | 21 | NO |
| 42 | taCasp3 | DG |  |  |  | 18 | 21 | NO |
| 43 | taCasp3 | DG |  |  |  | 8 | 13 | YES |
| 44 | taCasp3 | DG |  |  |  | 5 | 10 | YES |
| 45 | taCasp3 | DG |  |  |  | 10 | 9 | YES |
| 46 | taCasp3 | DG |  |  |  | 6 | 6 | YES |
| 47 | KA | Central |  | | | 0 | 21 | NO |
| 48 | KA | Central |  |  |  | 0 | 21 | NO |
| 49 | KA | Central |  |  |  | 5 | 21 | NO |
| 50 | KA | Central |  |  |  | 10 | 21 | NO |
| 51 | KA | Central |  |  |  | 18 | 21 | NO |
| 52 | KA | Central |  |  |  | 22 | 21 | NO |
| 53 | KA | Central |  |  |  | 23 | 21 | NO |
| 54 | KA | Central |  |  |  | 30 | 21 | NO |
| 55 | KA | Central |  |  |  | 15 | 17 | YES |
| 56 | KA | Central |  |  |  | 16 | 11 | YES |

Notes: KA, Kainic acid; DTA, Diphtheriatoxin A fragment; taCasp3, caspase3; CA1, cornu ammonis 1; CA3, cornu ammonis 3; DG, dentate gyrus.
