# Supplementary material for: Ablation of Unilateral Hippocampal GABAergic Neurons: A Novel Mouse Model of Mesial Temporal Lobe Epilepsy With Hippocampal Sclerosis
Source: CNS Neurosci Ther. 2026 Jan 31;32(2):e70772. doi: 10.1002/cns.70772 (PMC12859687; doi:10.1002/cns.70772)
Supplement: Supplementary file 1 — Appendix S1: cns70772‐sup‐0001‐Supinfo.zip. [file CNS-32-e70772-s001.zip › cns70772-sup-0002-FigureS1-S4@Supplementary Figure Legends.docx]

**Supplementary figure legends**

**Figure S1. Alterations of GABAergic neuron and glutamatergic neuron numbers in the hippocampal GABAergic neuron ablation mouse models. (A)** RNAscope in situ hybridization of VGLUT1 (Slc17a7) and VGAT (Slc32a1) at hippocampal subregions (CA1, DG, CA3) in AAV-DIO-taCasp3-TEVp-mCherry injection models at CA1 or DG subregion. Scale bar = 100 µm. **(B-C)** Statistical analysis of VGAT positive (B) and VGLUT1 positive (C) cell numbers in (A). n = 6 per group, one-way ANOVA followed by Tukey’s test. Data are presented as mean ± SD. ** p<0.01, **** p<0.0001, ns means not significant. VGLUT1, vesicular glutamate transporter 1; VGAT, vesicular GABA transporter.

**Figure S2. Alterations of GABAergic neuron signals in KA hippocampal injection models.** **(A**) Immunofluorescence detection of NeuN and VGAT expression at hippocampal subregions (CA1, DG, CA3) in KA injection models and NC. KA injected at central hippocampus. Scale bar = 100 µm. **(B)** Statistical analysis of VGAT positive signal ratio in (A). n = 6 per group, one-way ANOVA followed by Tukey’s test. Data are presented as mean ± SD. **** p<0.0001, ns means not significant. VGAT, vesicular GABA transporter; KA, kainic acid; NC, negative control.

**Figure S3. Alterations of excitatory neuron signals in KA hippocampal injection models.** **(A**) Immunofluorescence detection of NeuN and VGLUT expression at hippocampal subregions (CA1, DG, CA3) in KA injection models and NC. KA injected at central hippocampus. Scale bar = 100 µm. **(B-C)** Statistical analysis of VGLUT positive signal ratio (B) and NeuN positive cell numbers (C) in (A). n = 6 per group, one-way ANOVA followed by Tukey’s test. Data are presented as mean ± SD. *** p<0.001, **** p<0.0001, ns means not significant. VGLUT, vesicular glutamate transporter; KA, kainic acid; NC, negative control.

**Figure S4. Neuronal degeneration detection in** **the hippocampal GABAergic neuron ablation and KA hippocampal injection mouse models. (A-B)** Neuronal degeneration detection by Fluoro-Jade® C staining in the hippocampal GABAergic neuron ablation (A) and KA hippocampal injection mouse models (B). Scale bar = 100 µm. KA, kainic acid.
