# Supplementary figures and images for: Ablation of Unilateral Hippocampal GABAergic Neurons: A Novel Mouse Model of Mesial Temporal Lobe Epilepsy With Hippocampal Sclerosis
Source: CNS Neurosci Ther. 2026 Jan 31;32(2):e70772. doi: 10.1002/cns.70772 (PMC12859687; doi:10.1002/cns.70772)

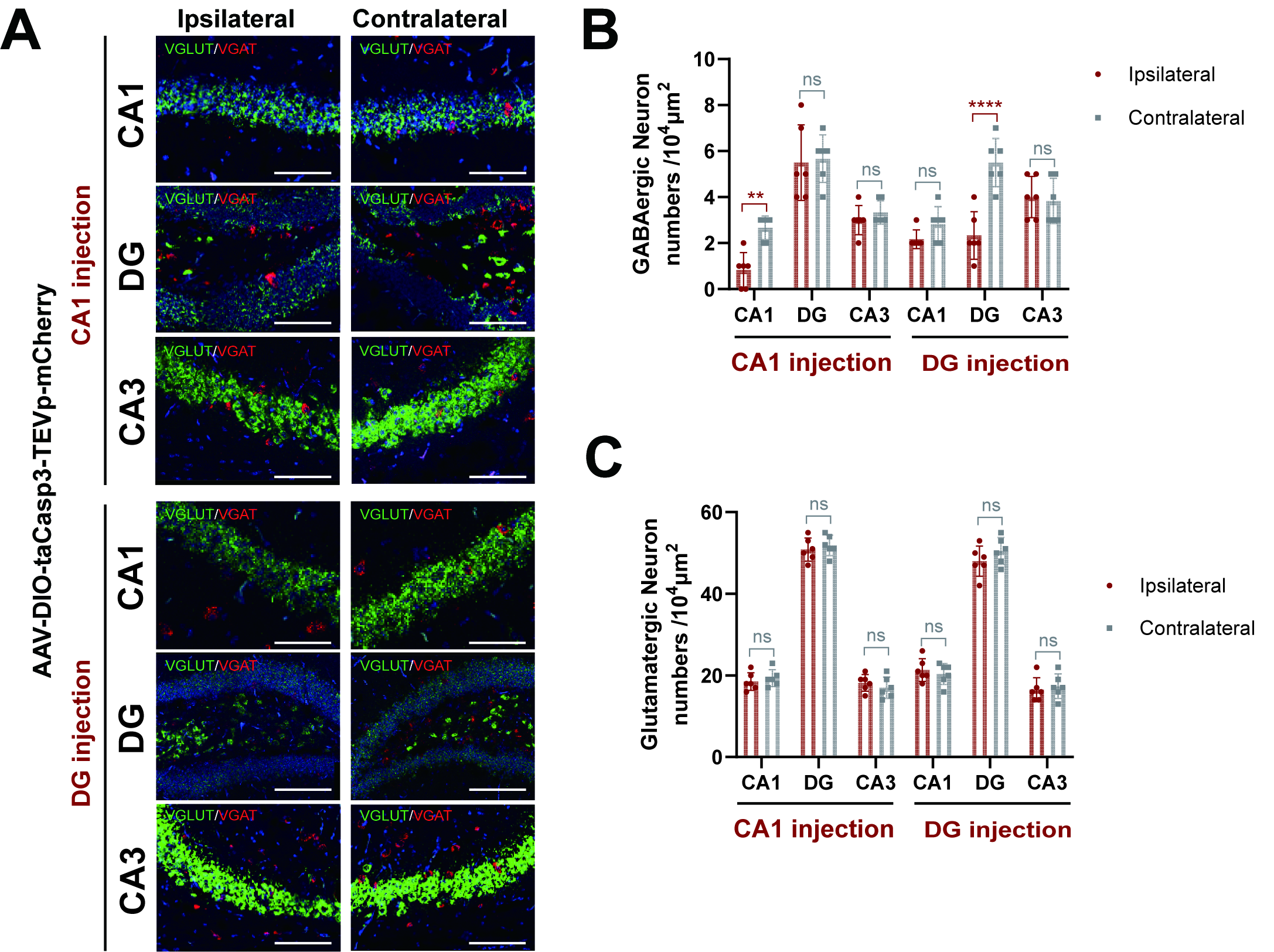

Supplement: Supplementary file 1 — Appendix S1: cns70772‐sup‐0001‐Supinfo.zip. [file CNS-32-e70772-s001.zip › cns70772-sup-0003-FigureS1@Supplementary Figure1.tif]

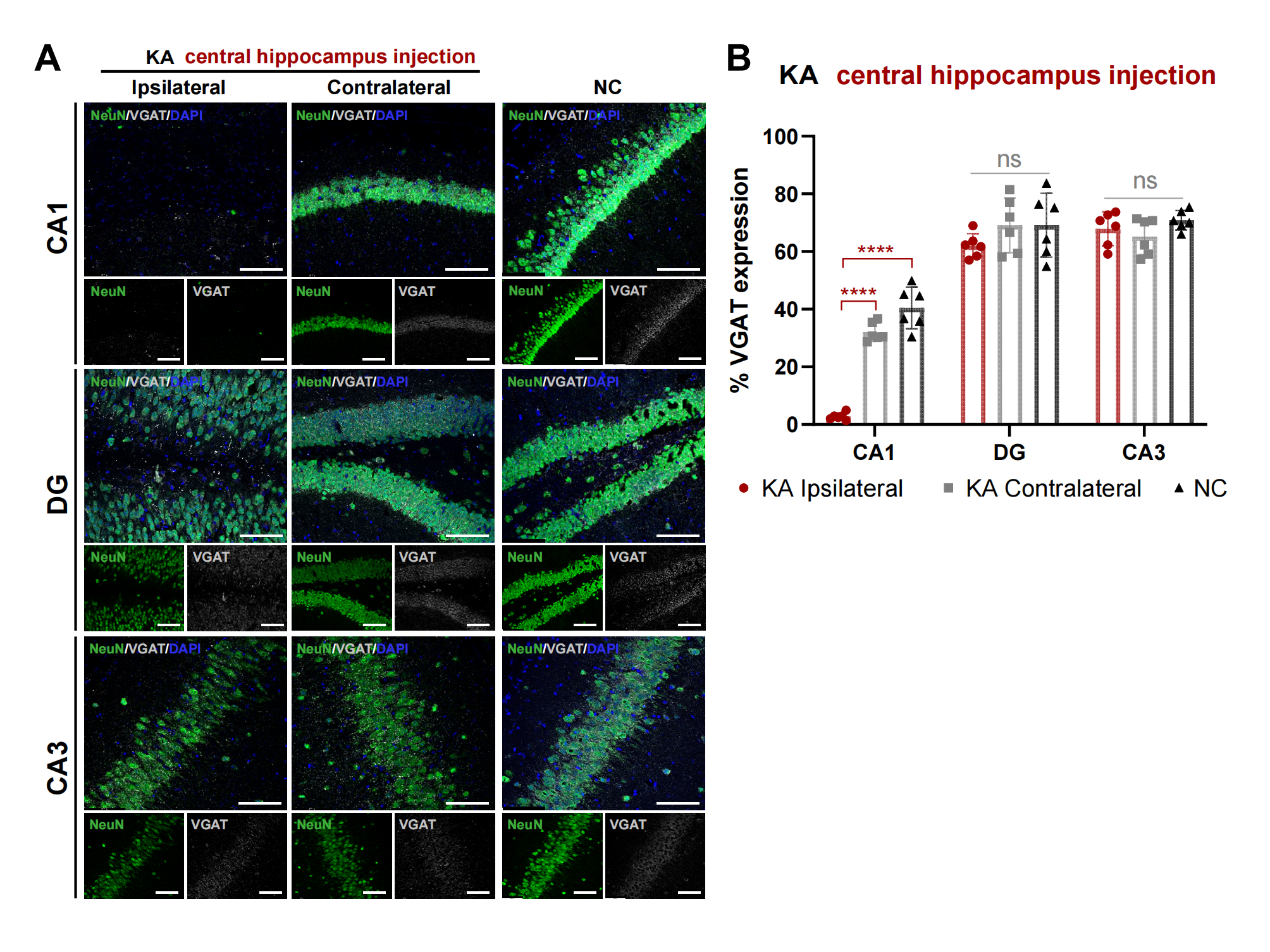

Supplement: Supplementary file 1 — Appendix S1: cns70772‐sup‐0001‐Supinfo.zip. [file CNS-32-e70772-s001.zip › cns70772-sup-0004-FigureS2@Supplementary Figure2.tif]

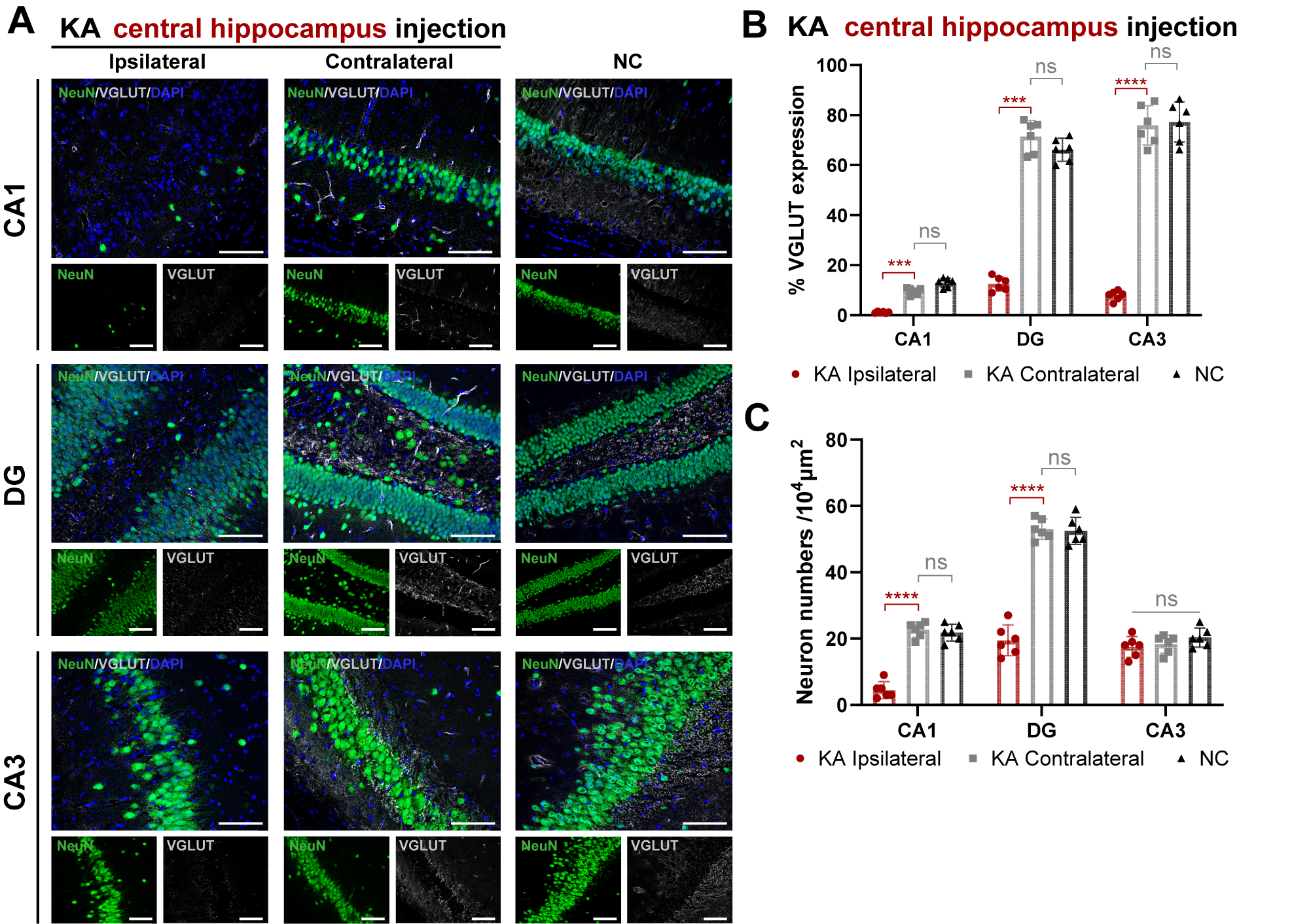

Supplement: Supplementary file 1 — Appendix S1: cns70772‐sup‐0001‐Supinfo.zip. [file CNS-32-e70772-s001.zip › cns70772-sup-0005-FigureS3@Supplementary Figure3.tif]

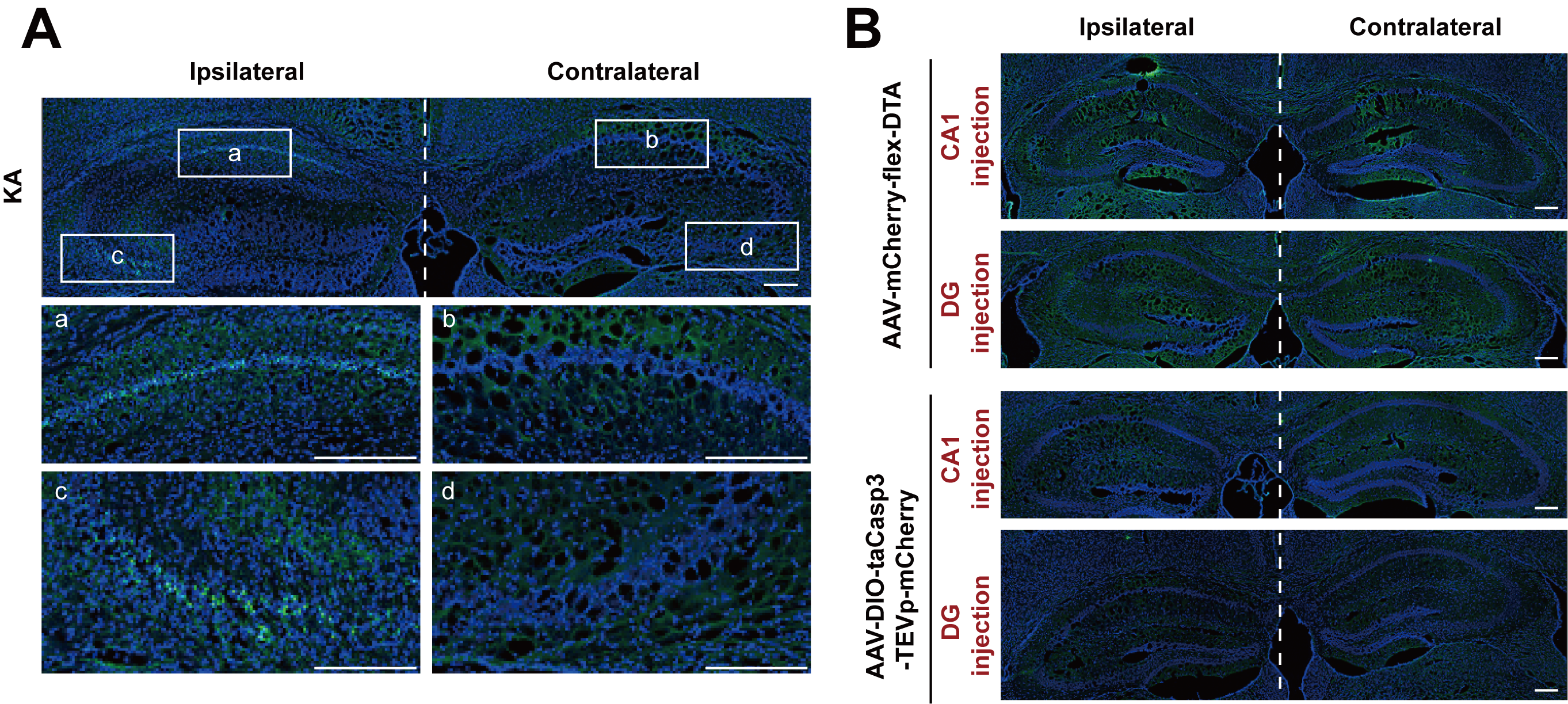

Supplement: Supplementary file 1 — Appendix S1: cns70772‐sup‐0001‐Supinfo.zip. [file CNS-32-e70772-s001.zip › cns70772-sup-0006-FigureS4@Supplementary Figure4 FJC.tif]
